# Cyclic Adenosine Monophosphate Signaling in Chronic Kidney Disease: Molecular Targets and Therapeutic Potentials

**DOI:** 10.3390/ijms25179441

**Published:** 2024-08-30

**Authors:** Charlotte Delrue, Reinhart Speeckaert, Rafael Noal Moresco, Marijn M. Speeckaert

**Affiliations:** 1 Department of Nephrology, Ghent University Hospital, 9000 Ghent, Belgium; charlotte.delrue@ugent.be; 2 Department of Dermatology, Ghent University Hospital, 9000 Ghent, Belgium; reinhart.speeckaert@ugent.be; 3 Graduate Program in Pharmaceutical Sciences, Center of Health Sciences, Federal University of Santa Maria, Santa Maria 97105-900, Brazil; rnmoresco@ufsm.br; 4 Research Foundation-Flanders (FWO), 1000 Brussels, Belgium

**Keywords:** cyclic adenosine monophosphate, chronic kidney disease

## Abstract

Chronic kidney disease (CKD) is characterized by a steady decline in kidney function and affects roughly 10% of the world’s population. This review focuses on the critical function of cyclic adenosine monophosphate (cAMP) signaling in CKD, specifically how it influences both protective and pathogenic processes in the kidney. cAMP, a critical secondary messenger, controls a variety of cellular functions, including transcription, metabolism, mitochondrial homeostasis, cell proliferation, and apoptosis. Its compartmentalization inside cellular microdomains ensures accurate signaling. In kidney physiology, cAMP is required for hormone-regulated activities, particularly in the collecting duct, where it promotes water reabsorption through vasopressin signaling. Several illnesses, including Fabry disease, renal cell carcinoma, nephrogenic diabetes insipidus, Bartter syndrome, Liddle syndrome, diabetic nephropathy, autosomal dominant polycystic kidney disease, and renal tubular acidosis, have been linked to dysfunction in the cAMP system. Both cAMP analogs and phosphodiesterase inhibitors have the potential to improve kidney function and reduce kidney damage. Future research should focus on developing targeted PDE inhibitors for the treatment of CKD.

## 1. Introduction

Chronic kidney disease (CKD) is a condition that gradually decreases kidney function over time. It affects roughly 10% of the world’s population and greatly raises rates of morbidity and mortality. The development of CKD is a complex process that involves a variety of cellular and molecular pathways, e.g., the 3′-5′-cyclic adenosine monophosphate (cAMP) signaling pathway, which controls transcription, metabolism, mitochondrial homeostasis, cell division, and cell death [1]. The evolutionary conservation of cAMP signaling, which extends from bacteria to mammals, emphasizes the importance of this signaling pathway [2,3]. Dysregulation of the cAMP pathway has been associated with numerous features of CKD, including glomerular hypertension, inflammation, and fibrosis [4]. As a result, pharmacological targeting of the cAMP signaling system appears to be a promising method to treating CKD. Modulating cAMP levels and activity with phosphodiesterase (PDE) inhibitors, adenylate cyclase (AC) activators, or cAMP analogs has shown promise in preventing kidney injury in preclinical models. This review seeks to offer a complete overview of the cAMP signaling in the context of CKD. We will describe the molecular targets of the cAMP pathway, their significance in the development of kidney disease, and the potential therapeutic advantages of modifying this system.

## 2. Methodology

This descriptive review was based on a search of the literature in the online PubMed Database, which was conducted by the authors to identify relevant studies published between 1990 and 2024. The search terms included “cAMP”, “chronic kidney disease”, “CKD”, and “eGFR”. The authors also searched the reference lists of the relevant articles to identify additional studies. The inclusion criteria for the studies were as follows: (1) original research articles published in peer-reviewed journals; (2) studies that investigated the association between cAMP and kidney function, kidney disease, or other clinical renal outcomes; and (3) studies that reported quantitative data on cAMP levels. The exclusion criteria were studies that were not written in English and did not have an abstract in English. The authors extracted data from the included studies, including the study design, sample size, patient characteristics, cAMP measurement methods, and outcomes.

## 3. cAMP: Formation, Distribution, and Regulation

**Figure 1:**
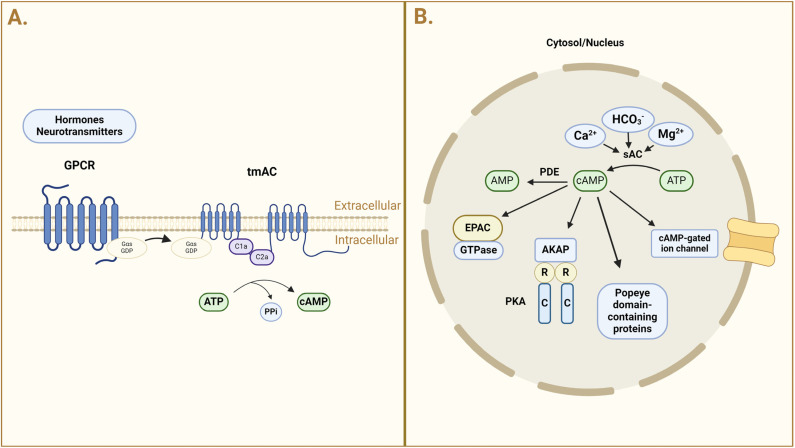
Schematic representation of cAMP signaling pathways. (**A**) The left section of the figure illustrates the activation of G protein-coupled receptors (GPCRs) by hormones and neurotransmitters, leading to the activation of transmembrane adenylyl cyclase (tmAC). Upon activation, the Gαs subunit exchanges GDP for GTP and interacts with tmAC, which then converts ATP into cyclic AMP (cAMP) and pyrophosphate (PPi). (**B**) Soluble adenylate cyclase (sAC) is involved in the synthesis of cAMP in the cytosol and nucleus and is activated by bicarbonate (HCO_3_^−^), calcium (Ca^2+^), and magnesium (Mg^2+^). Protein kinase A (PKA) can be activated by the produced cAMP by binding to its regulatory (R) subunits, which releases its catalytic (C) subunits in these compartments. The cAMP-gated ion channels in the cytosol can also be opened by cAMP, and exchange proteins directly activated by cAMP (Epac) can also activate GTPases. Moreover, A-kinase-anchoring proteins (AKAPs) and Popeye-domain-containing proteins interact with cAMP. AKAPs help to promote cAMP signaling in the cytosol and at the nuclear envelope.

The enzyme AC converts adenosine triphosphate (ATP) into cAMP, a secondary messenger. This conversion includes breaking the pyrophosphate link in ATP, which leads to the production of the cyclic structure of cAMP. Many key cellular functions, including transcription, metabolism, mitochondrial homeostasis, cell division, and cell death, are dependent on cAMP. Nevertheless, studies on cAMP signaling have revealed that cAMP is compartmentalized inside the cell, which aids in the selectivity and specificity of the signal [5]. Transmembrane AC (tmAC) generates cAMP primarily near the plasma membrane [3], whereas soluble AC (sAC) forms cAMP pools in various cellular compartments such as the cytosol, mitochondria, and nucleus [6,7]. TmACs are sensitive to G proteins, hence their activity is influenced by hormones and neurotransmitters (Figure 1).

Among the 800+ G protein-coupled receptors (GPCRs), some activate AC through the stimulatory G protein alpha subunit (Gαs), whereas others block certain ACs through the inhibitory G alpha subunit (Gαi/o). Indirect control of ACs occurs as a result of the activation of many signaling pathways. The most notable of these is calcium, which, when bound to calmodulin (CaM), may activate AC1 and AC8, while inhibiting (independent of CaM) AC5 and AC6. The C1b domain, which is C-terminally close to the catalytic site, regulates several ACs allosterically. Calcium-bound CaM activates AC1 and AC8 in this area, while Gβγ subunits can positively regulate AC2 by interacting with the C1b domain [3]. Once cAMP is generated, the downstream process can begin. There are four recognized downstream cAMP effectors in eukaryotic cells: protein kinase A (PKA), exchange protein directly activated by cAMP (Epac), cyclic nucleotide-gated channels, and Popeye-domain-containing proteins [4,5].

sAC is not responsive to G proteins but can be activated by bicarbonate (HCO_3_**^−^**), Mg^2+^, and Ca^2+^. The activation of sAC by bicarbonate anions is a hallmark feature, suggesting that it is important in metabolic activity detection [4]. Secondly, cAMP hydrolyzing phosphodiesterases (PDEs) help to compartmentalize cAMP signaling by being concentrated around cAMP production sites, restricting its diffusion to other compartments [8]. Finally, scaffolding proteins such as A-kinase-anchoring proteins (AKAPs) link cAMP production to functional effectors such as protein kinase A (PKA) and Epac. Several AKAPs, including AKAP18, AKAP220, AKAP-Lbc, and STIP1 homology and U-box-containing protein 1 (STUB1), coordinate signaling modules that control cAMP levels and PKA activity in collecting duct main cells [9,10,11,12]. These AKAPs assist cAMP localization by organizing multiprotein complexes known as signalosomes, assuring signal selectivity and efficiency. cAMP binds to PKA, causing it to activate and then phosphorylate target proteins. PKA phosphorylates aquaporin-2 (AQP2) during water reabsorption, which promotes its translocation to the plasma membrane. This mechanism is closely controlled by AKAPs, which guarantee that PKA is accurately targeted to AQP2-containing vesicles [13,14,15]. AKAPs also facilitate the formation of a signalosome that includes cyclin-dependent kinase 18 (CDK18), the E3 ubiquitin ligase STUB1 (also known as CHIP), PKA, and AQP2. This complex controls AQP2 location and abundance through cAMP-mediated phosphorylation and ubiquitination, which are critical for its proper function and regulation [16,17,18]. AKAPs can bind not just PKA, but also other kinases, ACs, PDEs, GPCRs, and phosphatases [5]. As a result, AKAPs, which are localized at particular subcellular locations, serve an important role in keeping cAMP signaling compartmentalized [3].

cAMP plays an important function in kidney physiology because its production and actions are highly localized and specific to particular nephron segments and receptors. In rats and rabbits, basal renal AC activity is higher in the medulla than in the cortex, showing that cAMP is differentially regulated in different areas of the kidney [19]. The distribution of different AC isoforms throughout the nephron supports a variety of cellular activities. Hormone-stimulated renal cAMP production is both site-dependent and receptor-specific. For example, the same hormone can induce cAMP synthesis in one nephron segment while inhibiting it in another, thereby enhancing the hormone’s functional versatility [20,21]. Key hormones such as calcitonin [22,23], dopamine [24,25], glucagon [26,27], glucagon-like peptide (GLP-1) [28,29], isoproterenol [22,30], parathyroid hormone (PTH) [31,32], arginine vasopressin (AVP) [31,33], and vasoactive intestinal peptide (VIP) [34] significantly increase cAMP formation in the kidney.

The cAMP signaling pathway is controlled by a feedback loop. PDEs’ degradation of cyclic nucleotides is a crucial pathway of rapidly lowering their cellular content while also controlling the intensity and length of their reactions by hydrolyzing the phosphodiester bond to generate 5′-AMP, which terminates the signal. Three of the eleven mammalian PDE families exclusively hydrolyze cAMP (PDE4, 7, and 8), whereas five (PDE1, 2, 3, 10, and 11) hydrolyze both cAMP and cyclic guanosine monophosphate (cGMP) [35].

## 4. cAMP in Kidney Physiology

### 4.1. cAMP and AVP in the Collecting Duct

The collecting duct is crucial for regulating water and electrolyte homeostasis, as well as acid–base balance [9]. AVP interacts with the V2 receptors (V2Rs) on the surface of main cells, causing a rise in cAMP via AC3/AC5/AC6 and then activating PKA. This cascade results in the redistribution of the water channel AQP2 from intracellular vesicles to the plasma membrane, thereby facilitating water reabsorption from the tubular fluid [36,37,38]. Water then enters the principal cells through the collecting duct lumen and exits via AQP3 and AQP4, always present in the basolateral plasma membrane [39,40]. In the primary cilia of inner medullary collecting duct cells, V2R activation by AVP also promotes a localized rise in cAMP, emphasizing the importance of local cAMP signaling for effective water reabsorption [37,41].

### 4.2. cAMP and Kidney Fibrosis

Kidney fibrosis is a critical element in the progression of CKD, characterized by excessive deposition of extracellular matrix (ECM) components, mainly collagen, which causes scarring and loss of kidney function, as well as podocyte death, inflammation, and monocyte infiltration [42]. cAMP is essential for intracellular signal transduction and has been linked to the development of kidney fibrosis. Fibrotic kidney tissues show lower amounts of cAMP. Restoration of cAMP levels can improve tubular atrophy and ECM deposition, principally by limiting the conversion of fibroblasts to myofibroblasts and lowering their proliferation and collagen production. PDE4, a particular phosphodiesterase, plays an important role in this situation. Inhibition of PDE4 by small interfering (si)RNA or specific inhibitors like rolipram reduces renal interstitial fibrosis and protects mitochondrial function in tubular epithelial cells [43].

Several cAMP-based signaling pathways have been linked to kidney fibrosis, including those mediated by nitric oxide/atrial natriuretic peptide (ANP)/guanylyl cyclases and cAMP-dependent PKA. These pathways control a variety of cellular activities related to fibrosis, including epithelial-to-mesenchymal transition (EMT), fibroblast proliferation, and ECM synthesis [44]. One critical aspect of the antifibrotic action of cAMP is its ability to blunt EMT, contributing to fibrosis [45]. cAMP can play a dual role in this process as it has both anti-fibrotic and fibrotic properties. Specialized pro-resolving mediators (SPM) are renoprotective and slow the advancement of CKD-associated fibrosis by promoting inflammation resolution [46,47]. The antifibrotic actions of SPM are dependent on intracellular cAMP levels. For example, SPM RvD1 activates the Gs-coupled GPCR N-formyl peptide receptor 2/lipoxin A4 receptor (FPR2/ALX), which raises cAMP levels in leukocytes and non-immune cells [48]. Furthermore, when *E. coli* causes inflammation in human macrophages, resolvin D1 (RvD1) and RvD5 reduce PDE4B and, hence, raise cAMP levels [49]. Lypoxines (LX), which are generated by neutrophils, platelets, and resident tissue immune cells at inflammatory areas, function as inflammation-breaking agents [47]. LXs may indirectly raise cAMP levels by activating purine receptors (for example, P2RY11) [50]. An increase in cAMP stimulates PKA, which phosphorylates cAMP response element-binding protein (CREB) and so activates Epac. CREB reduces transforming growth factor-beta (TGF-β)-mediated profibrotic gene transcription, while Epac inhibits Smad-dependent profibrotic gene transcription [44]. Similarly, cAMP increases with butaprost, a selective agonist of the Gs-coupled EP2 receptor, reduces kidney fibrosis in human kidney slices and unilateral ureteral blockage in mice [51]. Similarly, VIP promotes cAMP production in the RAW 264 macrophage cell line, and its administration to spontaneous hypertensive rats, a model of interstitial fibrosis without glomerulosclerosis, lowers fibrosis [34]. However, cAMP can also have fibrotic properties. Fibrosis is linked to an increase in extracellular adenosine levels. Kidney fibroblasts express AR2B, a Gs-coupled adenosine receptor. AR2B activation boosts cAMP signaling in cultured kidney NRK-49F fibroblasts, leading to a two- to four-fold rise in profibrotic and proinflammatory chemicals such as smooth muscle actin-alpha (SMA-α), interleukin (IL)-6, TGF-β, connective tissue growth factor (CTGF), and fibronectin [52]. Thus, distinct GPCRs and, most likely, different cAMP pools have opposing effects on the development of kidney fibrosis [53].

Understanding the major cAMP signaling compartments may lead to new methods for treating fibrosis in CKD. Given its critical role in fibrosis modulation, targeting the cAMP pathway is a possible therapeutic option. PDE inhibitors, such as rolipram, have shown promise in preclinical renal fibrosis models by increasing cAMP levels and demonstrating antifibrotic effects [43]. Using cAMP analogs or drugs that raise intracellular cAMP levels is another therapeutic option. These therapies limit fibroblast development, inhibit myofibroblast conversion, and accelerate fibroblast demise, all of which contribute to fibrosis reduction [44].

### 4.3. cAMP and Immune Modulation

Chronic inflammation is a common feature of CKD and plays an important role in disease progression and the development of comorbidities. The manipulation of cAMP levels has shown promise in decreasing inflammation by reducing pro-inflammatory responses while promoting anti-inflammatory cytokine synthesis [54,55]. Accumulation of cAMP and activation of PKA and Epac1/2 reduces the production of TNF-α and IL-1β, two NF-κB signaling inducers [56,57,58]. As a result, NF-κB-induced proinflammatory cytokines and signaling decrease [55,59]. Furthermore, an increase in cAMP reduces T cell activation, neutrophil responses to oxidative stress, and eosinophil migration [60]. An increase in cAMP levels lowers levels of proinflammatory chemokines such as chemokine (C-C motif) ligand 3 (CCL3), chemokine (C-X-C motif) ligand 1 (CXCL1), CCL2, CCL4, and CCL11 [55]. Chemokine receptors are typically Gi/0 coupled, which is critical for lowering cAMP levels during inflammation [58]. Increasing cAMP levels in neutrophils inhibits oxidative bursts generated by granulocyte–monocyte colony-stimulating factor [61]. However, high cAMP buildup can cause macrophages to generate proinflammatory chemokines in vitro [55]

cAMP signaling is crucial for regulating the activity of immune cells implicated in kidney disease. In diabetic kidney disease (DKD), immune cells such as macrophages invade the kidney, causing inflammation and fibrosis. Targeting cAMP pathways may help control immunological responses, perhaps lowering kidney damage [62].

cAMP signaling pathways play an important role in epigenetic regulation by controlling the activity of key enzymes involved in DNA methylation and histone modifications. When the cAMP–PKA pathway is active, CREB may undergo phosphorylation, which recruits histone acetyltransferases to promoters, including the CBP. This recruitment promotes histone acetylation, resulting in a more open chromatin shape and enhanced gene transcription [63].

### 4.4. cAMP in Nephrotic Syndrome

Nephrotic syndrome is a kidney condition characterized by excessive protein loss in urine, resulting in hypoalbuminemia, hyperlipidemia, and edema. Podocyte dysfunction is crucial to the development of nephrotic syndrome, and cAMP signaling has been demonstrated to have a major impact on podocyte function [9]. Podocytes are required to maintain the glomerular filtration barrier. cAMP signaling controls a wide range of physiological processes in podocytes, including cell adhesion, cytoskeletal dynamics, and damage response. Increased cAMP levels activate PKA and Epac, which enhance podocyte survival and function. These signaling pathways support the restoration of podocyte foot processes and the maintenance of the actin cytoskeleton, both of which are necessary for preserving the filtration barrier [64].

Proteinuria, which is a characteristic symptom of nephrotic syndrome, is closely linked to the dysfunction of podocytes. cAMP can impact the synthesis and activity of crucial proteins in the slit diaphragm, such as nephrin and podocin. In particular, aberrant cAMP signaling may interfere with nephrin’s distribution and phosphorylation, endangering the slit diaphragm’s integrity and raising the risk of proteinuria. Increasing cAMP levels reduces proteinuria and improves podocyte function in animal models of focal segmental glomerulosclerosis and minimal change illness [65].

Given the importance of cAMP in proteinuria and podocyte function, therapeutic approaches to nephrotic syndrome may include targeting the cAMP signaling system. Preclinical investigations have indicated that PDE inhibitors can limit cAMP breakdown, improving cAMP signaling and protecting podocytes. Furthermore, investigating the use of PKA and Epac agonists may be advantageous, since they activate cAMP-dependent pathways, stabilize podocyte function, and reduce proteinuria in patients with nephrotic syndrome [66].

PDE4, which is expressed by neutrophils, eosinophils, cytotoxic T lymphocytes, and macrophages, is important in the control of inflammation [55]. Inhibiting PDE4, and, thus, increasing cAMP, reduces the recruitment of inflammatory leukocytes to tissues and has already been used to treat inflammatory diseases such as chronic obstructive pulmonary disease (COPD), inflammatory bowel disease, and neuroinflammation [67,68]. Roflumilast, a PDE4 inhibitor, reduces cadmium-induced kidney damage and oxidative stress in rats by regulating NF-κB activation [69].

In normal conditions, extracellular ATP activates cAMP-dependent pathways via P2 receptors, influencing podocyte cytoskeleton remodeling and albumin permeability via RhoA signaling. Stimulation of the purinoceptor 4 receptor (P2Y4), which triggers PKA activation and phosphorylation, reduces RhoA signaling. Wang et al. [70] discovered that either overactivation or inhibition of RhoA causes podocyte damage, loss of foot processes, and albuminuria. This signaling axis serves as a renoprotective mechanism for the glomerular barrier, protecting it from oxidative stress while also maintaining an adequate energy balance. However, the AKAP1/Drp1 complex also results in enhanced ATP production with accompanying reduced RhoA activity via the P2Y4/PKA/RhoA pathway. A thorough knowledge of how AKAP1- and PKA-directed signaling compartments work may lead to new targets for improving physiological podocyte function in disorders such as CKD [71].

Finally, cAMP signaling is critical for regulating podocyte function and the pathophysiology of nephrotic syndrome. cAMP contributes to the glomerular filtration barrier’s integrity via regulating cytoskeletal movements, protein expression, and cell survival. Targeting cAMP pathways is a possible therapeutic option for managing nephrotic syndrome and its accompanying consequences.

This review also highlights the diverse roles of cAMP in various kidney diseases. Table 1 summarizes the key functions and therapeutic implications of cAMP signaling in these conditions.

## 5. cAMP in Chronic Kidney Disease

### 5.1. Autosomal Polycystic Kidney Disease

Hyperactivation of the AVP system has been implicated in the progression of autosomal dominant polycystic kidney disease (ADPKD), a genetic disorder characterized by the development of numerous fluid-filled cysts in the kidneys. A key player in this process is cAMP [73]. In ADPKD, binding of AVP to V2 receptors (V2R) in the kidney raises intracellular cAMP levels. Elevated cAMP promotes cystogenesis by increasing cell proliferation and fluid release into the cyst lumen. This mechanism is intensified as serum AVP levels rise with disease development, resulting in further cAMP increases and accelerated kidney destruction. Similarly to ADPKD, cAMP influences cyst development and growth in autosomal recessive polycystic kidney disease (ARPKD) [74].

Tolvaptan, a V2R antagonist, is approved for the treatment of ADPKD because it inhibits the cAMP spike generated by AVP. Tolvaptan effectively lowers cAMP levels and prevents the formation of renal cysts while preserving kidney function by inhibiting V2. Tolvaptan has therapeutic potential in the management of ADPKD by slowing the rate of renal volume rise and moderating the decline in kidney function, as demonstrated by clinical trials [72]. Researchers are looking into alternative possible therapies for the cAMP pathway in addition to tolvaptan. In individuals with ADPKD, cAMP levels can be lowered or its downstream effects can be mitigated to slow down the development and evolution of cysts [75].

### 5.2. Diabetic Nephropathy

Diabetic nephropathy is a main complication of diabetes mellitus characterized by albuminuria, decreased glomerular filtration rate (GFR), and an increased risk of end-stage kidney disease (ESKD). Hyperglycemia is an important risk factor for diabetic nephropathy. It induces CBP-mediated H3K9/14 hyperacetylation in gene promoters of renal mesangial cells. This includes the promoters of major profibrotic factors like TGF-β1, TGF-β3, and connective tissue growth factor (CTGF), which drive renal fibrogenesis. High glucose levels increase cAMP production and PKA activity, leading to increased phosphorylation of p65 and CREB, thus promoting the recruitment of CBP and RNA polymerase II to these promoters [63].

cAMP plays an important role in the pathophysiology of diabetic nephropathy via many signaling pathways. In diabetic nephropathy, reduced cAMP levels are frequently detected in fibrotic kidney tissues. Restoration of cAMP levels can improve tubular atrophy and extracellular matrix deposition, principally by limiting the conversion of fibroblasts to myofibroblasts, lowering their proliferation and collagen synthesis [43]. In diabetic nephropathy, the activation of E-type prostaglandin receptors (EP2 and EP4) seems to cause enhancement of cAMP production. AKAP 1, only expressed in podocytes, binds PKA to the cytosolic face of the outer mitochondrial membrane and controls mitochondrial fusion and fission via interacting with Drp1. Streptozotocin (STZ)-induced hyperglycemia in rats enhances Drp1 phosphorylation at Ser637 and recruitment to AKAP1. The AKAP1/Drp1 complex reduces mitochondrial membrane potential while increasing reactive oxygen metabolite formation and apoptosis resulting in podocyte loss. Furthermore, knockdown of the *AKAP1* gene protected the kidney against diabetes-induced kidney damage [76].

Given its relevance in the development of diabetic nephropathy, targeting the cAMP pathway could be a useful therapy strategy. Activation of the cAMP/PKA pathway provides protection against fibrogenic signals and inflammation. Studies on diabetic nephropathy models have shown that GLP-1 receptor agonists increase cAMP levels, reducing oxidative stress and inflammation in the kidneys. Renal oxidative stress, mesangial expansion, and albuminuria decrease with treatment with GLP-1 receptor agonists like liraglutide, mostly through activating the cAMP–PKA pathway [77]. Another treatment method is the use of PDE inhibitors. Compound A, a selective PDE4 inhibitor, has potential in diabetic nephropathy by boosting cAMP levels and demonstrating anti-inflammatory, anti-fibrotic, and antioxidant properties in diabetic mice models [78]. Furthermore, activation of Epac reduces tubulointerstitial inflammation in diabetic nephropathy. Epac activation via 8-pCPT-2′-O-Me-cAMP inhibits macrophage infiltration and inflammatory cytokine release, relieving tubulointerstitial fibrosis [79].

### 5.3. cAMP and Bartter Syndrome

Bartter syndrome is a family of autosomal recessive illnesses distinguished by a deficiency in salt reabsorption in the thick ascending limb of the loop of Henle. This causes a variety of clinical symptoms, including as hypokalemia, metabolic alkalosis, hypercalciuria, and, in some cases, nephrocalcinosis and growth retardation. Recent research has revealed the critical role of cAMP in regulating ion transport and its implications in Bartter syndrome. AC6 is especially crucial for controlling the synthesis, phosphorylation, and location of the Na^+^-K^+^-2Cl^−^ cotransporter (NKCC2) protein, which is required for salt reabsorption in the kidney [36]. AC6 knockout mice demonstrated a variety of physiological changes, including mild hypokalemia, alkalosis, modest urine Na^+^/K^+^ loss, lower blood pressure, and increased plasma renin concentration. These data indicate a mild salt-losing phenotype with clinical symptoms similar to Bartter syndrome [80].

cAMP regulates ion channels and transporters that are necessary for renal salt reabsorption. Mutations in the genes that encode these transporters cause disruptions in the cAMP signaling pathways in Bartter syndrome. Mutations in the *NKCC2* gene, for example, interfere with the cAMP-dependent regulation of the transporter, resulting in reduced salt and chloride reabsorption [81]. In Bartter syndrome, activation of the EP2 and EP4 receptors appears to increase cAMP synthesis. These receptors are engaged in the prostaglandin E2 (PGE2) signaling pathway, which is overexpressed in Bartter syndrome and contributes to the renal salt-wasting phenotype. Enhanced cAMP synthesis through EP2 and EP4 receptors exacerbates salt and chloride loss, resulting in the hallmark signs of Bartter syndrome [82].

The interaction between AKAP1 and PKA controls mitochondrial fusion and fission via Drp1. Streptozotocin (STZ)-induced hyperglycemia in rats enhances Drp1 phosphorylation at Ser637 and its recruitment to AKAP1. The AKAP1/Drp1 complex reduces mitochondrial membrane potential, while increasing reactive oxygen metabolite formation and apoptosis, resulting in podocyte loss. Knockdown of the *AKAP1* gene protects the kidney against diabetes-induced kidney damage, which has implications for understanding the mitochondrial dysfunction observed in Bartter syndrome [76].

### 5.4. cAMP in Liddle Syndrome

Liddle syndrome is a rare autosomal dominant disorder characterized by hypertension and hypokalemia. This condition is caused by mutations in either the β or γ subunits of the epithelial sodium channel (ENaC). Because of these mutations, the ENaC remains hyperactive all the time, increasing salt reabsorption and resulting in hypertension. cAMP has an important role in regulating ENaC trafficking and function, which is disrupted by ENaC mutations that cause Liddle syndrome. cAMP influences the regulation of ENaC via a variety of signaling mechanisms. Normally, cAMP stimulates ENaC function by promoting its translocation to the cell surface. The appropriate operation of ENaC in salt absorption depends on this translocation. Phosphorylating ENaC subunits facilitates their trafficking and insertion into the membrane, hence increasing the rate of ENaC translocation to the apical membrane of epithelial cells. Vesicle trafficking inhibition has demonstrated the importance of cAMP-mediated stimulation for the appropriate surface expression of ENaC. Liddle syndrome is characterized by a major disruption of this regulating system [83].

Mutations associated with Liddle syndrome often result in the deletion of the PY motif in the β or γ subunits of ENaC. This PY motif is essential for the binding of the ubiquitin ligase Nedd4-2, which normally ubiquitinates ENaC and targets it for degradation, thus reducing its surface expression. The loss of this motif due to mutations prevents Nedd4-2 from binding, leading to increased surface expression of ENaC and persistent sodium reabsorption. Consequently, the constitutive activation of ENaC in Liddle syndrome is partly due to a failure in the cAMP-mediated regulation of channel internalization and degradation [84]. cAMP is also involved in the proteolytic digestion of ENaC. Under normal conditions, cAMP stimulates the cleavage of ENaC subunits, which is required for complete activation. Mutations in Liddle syndrome boost ENaC surface expression while simultaneously altering its cleavage pattern, resulting in a greater fraction of fully functioning channels at the membrane. Excessive salt reabsorption, which contributes to hypertension in Liddle syndrome patients [85], is produced by both elevated surface expression and altered cleavage state. ENaC is controlled by the cAMP signaling pathway, and genetic defects diminish it, resulting in the pathophysiological symptoms of Liddle syndrome. The regulatory network that governs cAMP signaling, ENaC trafficking, and proteolytic processing is complicated, and genetic changes have a considerable impact on these activities [86].

### 5.5. cAMP in Renal Tubular Acidosis

Renal tubular acidosis (RTA) is a disorder in which the kidneys fail to adequately acidify urine, resulting in a systemic acid–base imbalance. RTA can be classified into several categories, the most frequent of which being distal RTA (dRTA). cAMP signaling in distal RTA regulates the acid-base balance via modulating proton pumps and bicarbonate transporters. cAMP modulates the function of proton pumps, specifically vacuolar-type H^+^-ATPase (V-ATPase), in the kidney’s distal tubules. When V-ATPase secretes protons into urine, it becomes more acidic and contributes to the maintenance of the systemic acid–base balance. V-ATPase moves to the apical membranes of intercalated cells in response to high cAMP levels, increasing proton secretion. Reduced proton secretion and systemic acidity are the outcomes of this pathway’s disruption in dRTA, which is crucial for normal urine acidification [87]. Bicarbonate transporters in the distal nephron are likewise impacted by cAMP. Maintaining the acid–base balance requires bicarbonate reabsorption, which is facilitated by the sodium bicarbonate cotransporter (NBCe1). cAMP signaling increases the activity and membrane expression of NBCe1, which promotes bicarbonate reabsorption. This control is critical in preventing metabolic acidosis by enabling efficient bicarbonate reabsorption from the filtrate into the circulation [36].

PDEs play an important function in controlling cAMP levels by facilitating its breakdown. PDE activity can cause a drop in cAMP levels, which can alter the signaling pathways in the distal nephron that controls the proton pump and bicarbonate transporters. Decreasing PDE activity increases cAMP levels, which leads to increased acid secretion and bicarbonate absorption. This method has potential therapeutic applications for controlling dRTA by restoring the equilibrium of acid–base regulation [43]. Epac proteins, which are guanine nucleotide exchange factors activated directly by cAMP, also play an important role in renal acid–base regulation. Epac2, in particular, has been linked to controlling acid secretion in the distal nephron. Studies on Epac2-deficient mice have revealed that they have defective urine acidification, emphasizing the necessity of Epac2 in maintaining correct acid–base balance. This shows that targeting Epac2 may be a viable treatment strategy for treating dRTA [88].

### 5.6. cAMP and Nephrogenic Diabetes Insipidus

In nephrogenic diabetes insipidus (NDI), the kidneys do not concentrate urine, causing excessive urination and thirst. This condition is caused by a deficiency in the reaction of the kidneys to the antidiuretic hormone vasopressin, whose effects are mediated via cAMP. The function of the kidneys to concentrate urine in NDI is compromised by deviations from typical cAMP signaling pathways [15]. Vasopressin increases cAMP synthesis via attaching to the V2 receptor on renal collecting duct cells and activating AC. This rise in cAMP levels promotes the phosphorylation and trafficking of AQP2 water channels to the apical membrane, resulting in increased urine concentration and water absorption. In NDI, mutations in the V2 receptor or AQP2 disrupt this signaling pathway, leading to impaired cAMP production and defective water reabsorption [89]. Increased activity of cAMP–PDE isozymes, particularly PDE-IV, in the renal collecting ducts of NDI patients leads to rapid degradation of cAMP, thereby preventing the accumulation necessary for AQP2 activation. The kidneys’ inability to react to vasopressin correctly is hampered by excessive PDE activity, which exacerbates the symptoms of NDI [90].

Vasopressin-stimulated cAMP signaling may be restored by pharmacochaperones that can correct mutant V2 receptor folding and trafficking. In patients with NDI, these medications can increase cAMP levels and enhance water absorption by stabilizing the receptor in a functional shape. By addressing the underlying molecular abnormalities, this method has the potential to treat congenital NDI [91]. Recent research has also revealed several adenylate cyclase isoforms that regulate cAMP production in response to vasopressin. For example, AC6 plays an important role in renal concentrating. Mice missing AC6 had impaired cAMP production and decreased AQP2 phosphorylation, which mirrored the defects observed in NDI [36].

### 5.7. cAMP in Fabry Disease

Fabry disease is an X-linked lysosomal storage illness characterized by a lack of the enzyme alpha-galactosidase A (α-Gal A). This deficit causes glycosphingolipids to accumulate in many tissues, including the kidneys, particularly globotriaosylceramide (Gb3). The role of cAMP in Fabry disease has been established as a major component influencing lysosomal storage problems that contribute to kidney failure [92]. The main regulator of lysosome activity is cAMP. This regulatory mechanism is essential for preserving lysosomal homeostasis and preventing abnormal substrate buildup, such as that seen in Gb3. Improving cAMP signaling can stimulate lysosomal activity and minimize substrate buildup in lysosomal storage diseases [54]. The kidneys are one of the main organs damaged by Fabry disease. Gb3 buildup in kidney cells causes gradual kidney damage, which manifests as proteinuria, reduced GFR, and, eventually, ESKD. cAMP signaling has a role in a variety of renal activities, including ion channel control, water balance, and stress responses. Dysregulation of cAMP pathways in Fabry disease worsens renal pathology by affecting these essential activities [93].

Understanding the involvement of cAMP in Fabry disease opens potentially new therapy options. Reducing the accumulation of Gb3 is the aim of current treatments, which include enzyme replacement therapy. However, addressing cAMP signaling pathways provides an alternative method to improve lysosomal function and postpone the onset of disease. Preclinical models have shown the potential of novel drugs that boost cAMP levels or activate its signaling, indicating that these strategies could improve patient outcomes and supplement current treatments [94].

### 5.8. The Role of cAMP in Renal Cell Carcinoma (RCC) and Implications for CKD

Renal cell carcinoma (RCC) is the most common kind of kidney cancer in adults. cAMP plays a crucial role in the pathophysiology and progression of RCC via a variety of pathways. RCC is characterized by the overexpression of CREB1, a proto-oncogenic transcription factor. CREB1 regulates three important phases in tumor development: cell migration, proliferation, and apoptosis. By interfering with these essential cellular processes, CREB1 downregulation prevents the growth of RCC tumors. Moreover, miR-10b-5p and miR-363-3p, which bind directly to the 3′-untranslated region of CREB1 mRNA and consequently decrease its mRNA and protein expression, regulate the expression of CREB1 [95]. The critical function of this protein in the survival and proliferation of RCC cells is highlighted by the regulation of CREB1 by cAMP. Depending on the situation, the cAMP signaling system can either promote or inhibit tumor growth. In the case of RCC, when PKA and its downstream effectors, such as CREB, are active, cAMP promotes the growth of tumor cells. These pathways control the transcription of genes essential for the survival and multiplication of cells. Tyrosine kinase inhibitors express anti-tumor actions in RCC, which are enhanced by PDE4 inhibition and raise cAMP levels, through downregulating the P38 mitogen-activated protein kinases (MAPK) pathway [96]. These results suggest that cAMP signaling modulation may be a useful treatment approach in RCC and possibly other forms of CKD where cell proliferation is a problem.

Erythropoietin (EPO) overproduction is one of the associated paraneoplastic phenomena of RCC. The ability of the tumor to make EPO on its own causes its release to be frequently increased in RCC. EPO is a glycoprotein hormone that controls the synthesis of red blood cells. cAMP is required for the elevated secretion of EPO by RCC. In a study using a human kidney cancer cell line, PDE inhibitors such as 3-isobutyl-1-methyl-xanthine (MIX) and cAMP significantly enhanced the release of EPO. This reaction was accompanied by a rapid release of EPO from a cell storage pool, suggesting the significance of cAMP-mediated signaling pathways in regulating EPO synthesis [97]. Furthermore, forskolin, an AC activator that increases cAMP levels, enhances EPO production in RCC cells and in culture media [98].

### 5.9. cAMP in AKI-to-CKD Transition

Acute kidney injury (AKI) is a frequent clinical condition marked by a swift decline in kidney function. In cases where recovery from AKI is incomplete, there is a heightened risk of progressing to CKD [99]. AKI often leads to persistent tubular damage, inflammation, and fibrosis, which are key features in the development of CKD. The cAMP signaling pathway, known for its diverse cellular functions, including regulation of inflammation, fibrosis, and cellular apoptosis, could significantly influence this transition. For example, alterations in cAMP levels could exacerbate inflammatory responses and promote fibrotic pathways, both of which are crucial in the progression from AKI to CKD. Despite this, the detailed mechanisms behind this transition are still not fully understood. Macrophages are crucial in both kidney damage and tissue repair, yet their specific role in the progression from AKI to CKD is not clearly defined [100]. cAMP might play a role in the progression from AKI to CKD. EP4, a key G protein-coupled receptor, regulates intracellular cAMP levels through its interaction with intracellular G proteins, which subsequently activates downstream extracellular signal-regulated kinase 1/2 pathways. In a mouse model of AKI-to-CKD transition induced by ischemia-reperfusion injury (IRI), the E-type prostaglandin receptor 4 (EP4) appears to be selectively activated in renal macrophages [99].

## 6. Therapies Targeting cAMP Signaling

Several therapeutic agents targeting cAMP signaling pathways have shown promise in treating various kidney diseases. Table 2 summarizes these agents, their mechanisms of action, and their effects on kidney function and disease progression.

### 6.1. Phosphodiesterase Inhibitors

Currently, only a few PDE inhibitors are used clinically to treat the pathophysiological dysregulation of cyclic nucleotide signaling in various disorders, including erectile dysfunction, pulmonary hypertension, acute refractory cardiac failure, intermittent claudication, and chronic obstructive pulmonary disease [109]. Among the signaling components of the cAMP axis, PDEs have been investigated the most as prospective targets for renal disorders and may have significant therapeutic potential in treating CKD.

Indeed, the non-selective PDE inhibitor pentoxifylline, when used with RAAS blockers, lowers GFR in type 2 diabetes patients with CKD and delays progression to ESKD [110]. Pentoxifylline alone improves the condition of rats with 5/6 subtotal nephrectomy (a CKD model) by reducing proteinuria, glomerulosclerosis, interstitial inflammation, and fibrosis [101]. Although therapy with non-selective PDE inhibitors shows promise, employing specific inhibitors may be more successful and have fewer negative effects. For example, inhibiting PDE3 with olprinone hydrochloride, a treatment for acute heart failure, reduced kidney dysfunction and the development of multiorgan dysfunction syndrome in a mouse model [111]. Cilostamide, another PDE3 inhibitor, reduces the production of reactive oxygen metabolites, exhibiting anti-inflammatory properties [112,113]. Cilostazol, a PDE3 inhibitor licensed for the treatment of intermittent claudication, prevents diabetes-induced glomerular hypertrophy and the activation of an inflammatory response, including increased ICAMs and VEGF levels in the kidney [102]. The therapeutic benefits of cilostazol are based on artery dilation and local blood pressure reduction, which may similarly lower blood pressure in renal arteries in CKD patients [114].

CKD is associated with reduced NO bioavailability. Therefore, the effects of PDE5 inhibitors on cGMP and NO levels have been investigated in CKD prevention and therapy [101,115,116,117]. For example, in mice with STZ-induced diabetes, sildenafil inhibited PDE5 and reduced the progression of diabetic nephropathy and associated hypertension by improving hemodynamic parameters [103]. Another PDE5 inhibitor, PF-00489791, has shown nephroprotective effects in clinical phase II studies. PF-00489791, in conjunction with RAAS blockers, decreases albuminuria and the urine albumin-to-creatinine ratio in diabetic nephropathy [118]. The effects of PDE5 inhibition must be evaluated in the context of cGMP–cAMP interaction. Inhibiting PDE5 increases cGMP levels, which inhibits PDE3. While the affinity of PDE3 for cAMP and cGMP is comparable (Km values range from 0.1–0.8 μmol/L), the Vmax for cAMP is 4–10 times greater than that for cGMP [104]. Therefore, PDE3 inhibition is likely a mediator of the renoprotective actions of PDE5 drugs.

Some kidney diseases require reducing cAMP levels and can be treated with specialized PDE activators. A recent example is the creation of MR-L2, a small molecule used to treat polycystic kidney disease. This molecule activates long forms of PDE4 and suppresses cyst development in Madin-Darby canine kidney (MDCK) cells and primary cell cultures from PKD patients [105]. Hyperactivation of PDE3A may also have a renoprotective effect by reducing cAMP levels, as seen in hypertensive patients with hyperactivated PDE3A but no hypertensive kidney injury [119,120,121,122,123].

Overall, these examples demonstrate that employing PDE modulators might be a potential approach to CKD therapy. However, current inhibitors target all isoforms of a PDE family (Table 3) rather than individual isoforms. For example, milrinone inhibits both PDE3A and PDE3B [109]. The poor specificity of existing PDE modulators, given the extensive expression of PDE family members, is believed to cause negative effects from interfering with PDE action. Specificity and simultaneous control of local cAMP levels in specific cellular compartments can be achieved by focusing on defined PDE protein interactions, such as PDE-AKAP interactions [124]. New PDE4 activators are an improvement, as they solely target the PDE4 family’s long isoforms [105].

### 6.2. Adenylate Cyclase Activators

AC activators have a significant impact on cAMP levels within kidney cells, affecting a wide range of cellular functions essential to kidney function. AC activators enhance cAMP production, which modulates cell signaling, proliferation, differentiation, and apoptosis, all of which play essential roles in CKD regulation. To boost treatment efficacy, current discoveries in AC activators have focused on specific signaling pathways. As an example, the well-known AC activator forskolin has been the focus of extensive research due to its ability to increase cAMP levels in kidney cells, hence improving protective cellular responses and decreasing fibrosis [106]. Furthermore, new AC activators are being created to modulate cAMP synthesis more precisely, minimizing side effects and enhancing CKD treatment effectiveness. Pituitary adenylate cyclase-activating polypeptide (PACAP38) is one such novel activator that has demonstrated potential in preventing kidney injury through inhibition of the nuclear factor kappa-light-chain-enhancer of activated B cells (NF-κB) and suppression of proinflammatory cytokine production [107].

Structural modifications have been made to AC activators to improve their specificity. This makes it possible to activate different AC isoforms selectively, which is crucial for minimizing adverse effects and enhancing treatment accuracy. Particularly, AC6 has been identified as an important isoform in controlling cAMP levels in the kidney’s inner medulla. This regulation influences water reabsorption and sodium balance [36]. In studies on animal models, increasing cAMP signaling via AC activation enhanced kidney function and decreased disease development in CKD [132,133]. These findings have set the stage for clinical trials to assess the efficacy and safety of AC activators in CKD patients.

### 6.3. cAMP Analogs

Recent advancements in the production process of cAMP analogs have made it possible to precisely target renal cells and renal signaling pathways. These state-of-the-art cAMP analogs aim to enhance therapeutic outcomes by specifically influencing cellular pathways and reducing the negative effects associated with general cAMP control. The therapeutic potential of novel cAMP analogs has been increased by their ability to selectively activate or inhibit certain signaling pathways in kidney cells. For example, the use of PDE inhibitors like rolipram has shown promise in the treatment of renal fibrosis. Rolipram is a compound that raises cAMP levels and stimulates the Epac1/Rap1 pathway. It accomplishes this by directly suppressing PDE4, which is critical for minimizing fibrosis and protecting tubular epithelial cells. In tubular epithelial cells, cAMP signaling regulates the formation of mitochondria, which is required to maintain cellular energy balance and prevent fibrosis. Rolipram increases mitochondrial activity and structure in fibrotic circumstances by inhibiting the cAMP pathway. It restores the synthesis of peroxisome proliferator-activated receptor gamma coactivator 1-alpha (PGC-1α) and CCAAT/enhancer binding protein-beta (C/EBP-β) [43].

The discovery of drugs that may selectively activate or inhibit specific PKA isoforms or other cAMP-binding proteins represents a significant advancement in the field of cAMP analogs. This level of precision reduces off-target activity and enables tailored therapy outcomes. In cells that largely express this type of kinase, (Rp)-8-Br-cAMPS and (Rp)-8-Cl-cAMPS are efficient cAMP antagonists that primarily inhibit cAMP-dependent protein kinase I [108]. The compartmentalization of cAMP signaling inside cells is critical to the specificity of its effects. cAMP analogs capable of selectively modulating these segregated signals have been developed to improve therapeutic precision. PDE4D, for example, controls AQP2 trafficking in renal main cells, hence regulating vasopressin-mediated water reabsorption compartmentally. By targeting this specific mechanism, new PDE4 inhibitors can improve water reabsorption and treat disorders such as nephrogenic diabetic insipidus [134].

## 7. Conclusions and Future Perspectives

cAMP signaling is a key component in the pathophysiology of CKD. As a secondary messenger, cAMP controls several cellular functions such as fibrosis, inflammation, and cellular homeostasis that are critical for kidney function. The various mechanisms via which cAMP signaling affects kidney physiology and pathophysiology highlight the dual roles that it plays in both encouraging protective responses and accelerating the course of disease. CKD can be treated by reducing cAMP levels using analogues or activators, such as GLP-1 receptor agonists [135]. Combining RAAS inhibitors with cAMP modulators may enhance the effectiveness of CKD treatments. Given the intricate nature of cAMP signaling, a comprehensive therapeutic approach that takes into account various cellular contexts and disease stages is necessary.

To improve our knowledge of, and ability to treat, CKD via cAMP signaling, future research should concentrate on the creation of highly selective PDE inhibitors. These inhibitors should target isoforms specifically associated with kidney pathology, and may enhance therapeutic efficacy while reducing adverse effects. Additionally, it is crucial to comprehend how kidney cells compartmentalize cAMP signaling and devise methods to modify cAMP pools in specific subcellular areas. Clinical trials should be conducted to investigate the safety and effectiveness of combining cAMP modulators with other treatments, such as RAAS inhibitors or anti-inflammatory drugs, as these combinations may synergistically benefit CKD patients.

A better understanding of the genetic and molecular mechanisms underlying individual responses to cAMP-targeted medicines may accelerate the development of more personalized treatment programs. It may be able to predict an individual’s reaction to cAMP modulators by identifying biomarkers. Additionally, discovering new molecular targets in the cAMP signaling system that aid in the progression of CKD may lead to novel therapy options. Investigating how scaffolding proteins such as AKAPs operate in cAMP signaling may introduce new treatment strategies. Addressing specificity, compartmentalization, and customized approaches is crucial to developing more effective and tailored treatments.

## Figures and Tables

**Table 1:** Role of cAMP in various kidney diseases.

Kidney Disease	Role of cAMP	References
Nephrotic syndrome	cAMP modulates podocyte function, affecting cytoskeletal dynamics, cell adhesion, and protein expression crucial for maintaining the filtration barrier.	[9,64]
Diabetic nephropathy	cAMP influences the CREB-binding protein-mediated hyperacetylation of profibrotic genes, contributing to fibrogenesis and kidney damage.	[63]
Autosomal dominant polycystic kidney disease	cAMP promotes cystogenesis by stimulating cell proliferation and fluid secretion in the cyst lumen through V2 receptor activation.	[72]
Bartter syndrome	cAMP modulates ion transport and salt reabsorption in the thick ascending limb of the loop of Henle, impacting NKCC2 regulation.	[36]
Liddle syndrome	cAMP regulates the ENaC activity and trafficking, with mutations in ENaC disrupting this regulation and causing hypertension.	[55]
Renal tubular acidosis	cAMP affects proton pumps and bicarbonate transporters, regulating acid–base balance in distal tubules and influencing V-ATPase activity.	[16]
Nephrogenic diabetes insipidus	cAMP mediates vasopressin effects on water reabsorption by promoting AQP2 trafficking to the apical membrane in the collecting ducts.	[15]
Fabry disease	cAMP impacts lysosomal function and substrate accumulation, contributing to kidney dysfunction by modulating lysosomal enzyme activity.	[8]
Renal cell carcinoma	cAMP influences cell proliferation, migration, and apoptosis through CREB1 regulation, impacting tumor growth and survival.	[35]

Abbreviations: cAMP, cyclic adenosine monophosphate; CREB, cAMP response element-binding protein; NKCC2, Na^+^-K^+^-2Cl^−^ cotransporter 2; ENaC, epithelial sodium channel; AQP2, aquaporin-2.

**Table 2:** Therapeutic approaches targeting cAMP signaling in kidney diseases.

Therapeutic Agent	Status	Mechanism of Action	Kidney Disease	Effects on Kidney Function	References
Pentoxifylline	On the market (off label use)	Non-selective PDE inhibitor, increases cAMP levels	Diabetic nephropathy, CKD	Reduces proteinuria, inflammation, and fibrosis; delays progression to ESKD	[101]
Cilostazol	On the market (off label use)	PDE3 inhibitor, increases cAMP levels	Diabetic nephropathy, CKD	Prevents glomerular hypertrophy and inflammation; improves blood pressure and vascular function	[102]
Sildenafil	On the market (off label use)	PDE5 inhibitor, increases cGMP levels	Diabetic nephropathy	Reduces progression of nephropathy and hypertension; improves hemodynamic parameters	[103]
PF-00489791	Clinical trials	PDE5 inhibitor, increases cGMP levels	Diabetic nephropathy	Decreases albuminuria and urine albumin-to-creatinine ratio	[104]
GLP-1 Receptor Agonists	On the market	Activates cAMP production via GLP-1 receptor stimulation	Diabetic nephropathy	Reduces inflammation, oxidative stress, and fibrosis; improves renal function	[77]
MR-L2	Research stage	Activates long forms of PDE4, reduces cAMP levels	Polycystic kidney disease	Suppresses cyst development and progression	[105]
Forskolin	On the market	AC activator, increases cAMP levels	CKD	Promotes protective cellular responses, reduces fibrosis	[106]
PACAP38	Research stage	AC activator, reduces off-target effects	CKD	Prevents renal injury by suppressing proinflammatory cytokine production, inhibits p38 MAPK and NF-κB pathways	[107]
(Rp)-8-Br-cAMPS, (Rp)-8-Cl-cAMPS	Research stage	cAMP analogs, selectively inhibit cAMP-dependent protein kinase I	CKD	Target specific signaling pathways, reduce off-target actions	[108]
Rolipram	Research stage	PDE4 inhibitor, increases cAMP levels	Renal fibrosis	Activates Epac1/Rap1 pathway, reduces tubular epithelial cell damage and fibrosis	[43]

Abbreviations: cAMP, cyclic adenosine monophosphate; CKD, chronic kidney disease; ESKD, end-stage kidney disease; PDE, phosphodiesterase; cGMP, cyclic guanosine monophosphate; GLP-1, glucagon-like peptide-1.

**Table 3:** Overview of the different PDE isoforms, their localization in the kidney, and their role in CKD.

PDE Isoform	Localization in the Kidney	Role in CKD	References
PDE1 (cAMP) PDE1 (cGMP)	Cortical tubules + Proximal tubule epithelial cells +/++ Inner medullary collecting duct cells ++ Glomeruli ++++ Mesangial cells +++ Cortical tubules +++ Proximal tubule epithelial cells +/++ Inner medullary collecting duct cells +	Inhibition of PDE1 causes greater stimulation of ERK and proliferation of ADPKD cells.	[113,125]
PDE2	Glomeruli ++++ Proximal tubule epithelial cells +	cGMP-stimulated PDE2 mediates the inhibitory effect of nitric oxide on NaCl absorption by the medullary thick ascending limb of the loop of Henle.	[113,126]
PDE3	Glomeruli ++ Glomerular epithelial cells +/− Mesangial cells +++ Cortical tubules ++ Proximal tubule epithelial cells +/− Inner medullary collecting duct cells +	PDE3-linked cAMP–PKA pathway modulates mitogenesis.	[113,127,128]
PDE4	Glomeruli ++ Glomerular epithelial cells ++++ Mesangial cells ++++ Cortical tubules +++ Proximal tubule epithelial cells ++++ Inner medullary collecting duct cells +++	PDE4 inhibition suppresses oxidative stress, fibrosis, and inflammation in mesangial cells and podocyte cells, which protect podocyte loss, which leads to albuminuria and glomerulosclerosis. In tubulointerstitial lesions, PDE4 inhibition suppresses inflammation and epithelial–mesenchymal transition, which leads to tubulointerstitial fibrosis. PDE4-linked cAMP–PKA pathway modulates generation of reactive oxygen species.	[113,128,129,130]
PDE5	Glomeruli +++ Mesangial cells +++ Cortical tubules +++ Proximal tubule epithelial cells +/++ Inner medullary collecting duct cells +	PDE5 inhibition regulates the excretory function and hemodynamics of the kidney. PDE5 contributes to the regulation of renal vascular blood flow by limiting the vascular relaxation caused by cGMP. PDE5 contributes to the regulation of natriuresis through the degradation of cGMP.	[113,131]

Abbreviations. PDE: phosphodiesterases; CKD: chronic kidney disease; ERK: extracellular signal-regulated kinase; ADPKD: autosomal dominant polycystic kidney disease; cAMP: cyclic adenosine monophosphate; cGMP: cyclic guanosine monophosphate; PKA: protein kinase A; +/−: variable expression; +: low expression; ++: moderate expression, +++: high expression, ++++: very high expression.
